# Development and Validation of a Fully GMP-Compliant Process for Manufacturing Stromal Vascular Fraction: A Cost-Effective Alternative to Automated Methods

**DOI:** 10.3390/cells9102158

**Published:** 2020-09-24

**Authors:** Pauline François, Laurent Giraudo, Julie Veran, Baptiste Bertrand, Chloé Dumoulin, Houssein Aboudou, Fanny Grimaud, Marie Vogtensperger, Mélanie Velier, Laurent Arnaud, Luc Lyonnet, Stéphanie Simoncini, Benjamin Guillet, Françoise Dignat-George, Jérémy Magalon, Florence Sabatier

**Affiliations:** 1 Cell Therapy Department, Hôpital de la Conception, AP-HM, INSERM CIC BT 1409, 13005 Marseille, France; pauline.francois@ap-hm.fr (P.F.); Laurent.GIRAUDO@ap-hm.fr (L.G.); julie.veran@ap-hm.fr (J.V.); chloe.dumoulin@ap-hm.fr (C.D.); houssein.aboudou@ap-hm.fr (H.A.); fanny.grimaud@ap-hm.fr (F.G.); florence.sabatier@ap-hm.fr (F.S.); 2 Aix Marseille University, INSERM, INRAE, C2VN, 13005 Marseille, France; melanie.velier@ap-hm.fr (M.V.); stephanie.simoncini@univ-amu.fr (S.S.); benjamin.guillet@univ-amu.fr (B.G.); francoise.dignat-george@univ-amu.fr (F.D.-G.); 3 Plastic Surgery Department, Hôpital de la Conception, AP-HM, 13005 Marseille, France; baptiste.bertrand@ap-hm.fr; 4 French Military Blood Institute, 92140 Clamart, France; marie.vogtensperger@intradef.gouv.fr; 5 Laboratory of Hematology and Vascular Biology, Hôpital de la Conception, AP-HM, 13005 Marseille, France; laurent.arnaud@ap-hm.fr (L.A.); luc.lyonnet@ap-hm.fr (L.L.); 6 CERIMED, Aix Marseille University, AP-HM, 13005 Marseille, France

**Keywords:** stromal vascular fraction, GMP-compliant manufacturing, cell therapy, wound healing

## Abstract

The therapeutic use of adipose-derived stromal vascular fraction (SVF) is expanding in multiple pathologies. Various processes have been proposed for manufacturing SVF but they must be revisited based on advanced therapy medicinal product (ATMP) regulations. We report here the development and validation of a fully good manufacturing practices (GMP)-compliant protocol for the isolation of SVF. Adipose tissue was collected from healthy volunteers undergoing lipoaspiration. The optimal conditions of collagenase digestion and washing were determined based on measurements of SVF cell viability, yield recovery, and cell subset distribution. Comparability of the SVF obtained using the newly developed manufacturing process (n = 6) and the Celution-based automated method (n = 33), used as a reference, was established using inter-donor analyses. Characteristics of SVF (n = 5) generated using both manufacturing protocols were analyzed for an intra-donor comparison. In addition, these comparisons also included the determination of colony-forming unit fibroblast frequency, in vitro angiogenic activity, and in vivo regenerative effects in a mouse ischemic cutaneous wound model. We successfully developed a process for the generation of SVF presenting higher cell viability and yield recovery compared to the Celution device-based protocol. Characteristics of the SVF including phenotype, capacity for angiogenesis, and wound-healing promotion attested to the comparability of the two manufacturing processes. We validated an optimized non-automated process that should allow for a GMP-compliant, more affordable, and reduced-cost strategy to exploit the potential of SVF-based regenerative therapies.

## 1. Introduction

Adipose tissue (AT) has long been known for its filling ability, and autologous fat transfer, also called lipofilling, is performed daily to correct soft tissue defects [1,2]. AT is also a major source of multipotent mesenchymal stem/stromal cells (MSCs) [3,4]. These adipose-derived stem/stromal cells (ASCs) are not only used for fat engraftment but are also considered a promising product in the field of cell-based therapies. ASCs can differentiate into various cell types (e.g., smooth muscle cells [5], chondrogenic cells [6], endothelial cells [7,8], adipocytes [9]) and display significant paracrine activity through the secretion of a large panel of cytokines, chemokines, and growth factors responsible for regenerative and immunomodulatory effects [10,11,12,13]. However, the therapeutic use of ASCs requires an ex vivo expansion step that takes several weeks [14]. This limitation can be circumvented by using the stromal vascular fraction (SVF), which is a heterogeneous cell population containing 15–30% ASCs [15] obtained from the enzymatic digestion of AT. In addition to ASCs, SVF is composed of endothelial progenitor cells, pericytes, and hematopoietic cells [15]. These different cell subsets recapitulate the composition of AT microvasculature and have been shown to synergistically exert angiogenic, immunomodulatory, and anti-fibrosis effects that could be of interest in several pathologies [16,17,18] and are now the subject of a large number of clinical studies [19]. Thus, for example, SVF may participate in the treatment of inflammation, fibrosis, and vasculopathy in systemic sclerosis [20,21]. Studies have also used SVF in musculoskeletal disease with long-term positive results in knee osteoarthritis [22] or recalcitrant Achilles tendinopathy compared to control groups [23]. SVF has also been used in more invasive procedures such as intramyocardial injection in patients who underwent chronic ischemic cardiomyopathy with a good safety profile and significant functional improvement [24].

From a regulatory point of view, autologous SVF is considered an advanced therapy medicinal product (ATMP) according to the European Directive No. 1394/2007, as it is prepared using enzymatic digestion (substantial manipulation) and not intended to be used for the same essential function in the recipient and the donor (non-homologous use). Thus, since it is considered a drug product, SVF manufacturing must strictly follow good manufacturing practices (GMP) from its development to the markering authorization. In others words, appropriate measures and arrangements must be applied to safeguard the quality and the security of the product for their intended use. It included trained personnel, qualified premises and equipment, appropriate documentation, quality of production operations, quality of starting and raw materials, validated quality control and analytical methods, and a qualified person for the batch release. Common steps of SVF manufacturing include AT harvesting and washing, enzymatic digestion, centrifugation, and SVF pellet isolation and washing [9], as originally described by Zuk in 2001 [25]. However, to date, there is no consensus on the precise conditions attached to each stage of the process, even though variations in SVF production methods could impact the final cell composition. Altogether, this strengthens the need for the better standardization of SVF isolation processes and further comparability studies before implementation of an SVF manufacturing process intended for clinical use.

In this context, we aimed to develop an effective GMP-compliant and cost-optimized method to isolate therapeutic SVF. This study details the developpement of the standardized method, the validation of critical production parameters, and finally provides in vitro and in vivo comparability data on the manually isolated SVF with reference to SVF obtained from the most commonly used automated method.

## 2. Materials and Methods

### 2.1. Donor Specifications 

AT was obtained as excedent surgical residue from healthy donors undergoing liposuction for aesthetic purposes in the department of plastic surgery, La Conception University Hospital, Marseille, France. The study was conducted in accordance with the declaration of Helsinki and all subjects provided informed consent for scientific use of the surgical residues through a survey that was validated by the regulatory affairs department of AP-HM hospitals. AT samples were anonymized before transmission to the cell therapy unit.

### 2.2. AT Harvesting 

AT harvesting was performed by experienced surgeons from the plastic surgery department of La Conception university hospital in an operating room under general anesthesia after a standardized skin aseptic preparation using a Khouri cannula (Khouri Harvester, Koume, Lipoplasty Products, Sunrise, FL, USA), a 12-gauge 12-hole multiperforated cannula, through a closed circuit, preventing contamination of the harvested product. Infiltration was performed using a mixture of xylocaine 10 mg/mL and adrenaline 0.05 mg/mL diluted in a saline solution (NaCl; Sodium chloride 0.9% Viaflo, Baxter, Deerfield, IL, USA). The mean volume of harvested AT was 187.74 mL ± 68.28 mL. The AT was packaged in a medical device (transfer bag; Easyflex+, Macopharma, Mouvaux, France). Once harvesting was complete, the bag was immediately transported to the cell therapy unit.

### 2.3. Development of the LG SVF Isolation Process

The developed SVF production process, named the LG process after the name of the person who originally developed it, was conducted in a controlled cleanroom in accordance with GMP guidelines effective for the French hospital exemption for ATMP. Most stages were performed in a sterile, closed fluid circuit in a class B cleanroom, except for open steps requiring grade A laminar airflow. All consumables and materials were marketed for cleanroom use by suppliers and validated by the person in charge of the quality control of the GMP facility.

First, under laminar airflow, the harvested AT contained in a MACO bag (Macopharma) was transferred aseptically into a 250-mL Puregraft bag (Bimini Health Technologies, Solana Beach, CA, USA) using 60-mL syringes (Terumo, Tokyo, Japan). AT was washed three times, with a volume of lactate Ringer’s solution (RL; Ringer Lactate Viaflo; Baxter) equivalent to half of the total Puregraft volume, preheated at 37 °C, using the Puregraft device, allowing the drainage of residual wetting/tumescent solution, red blood cells, and other debris. After discarding the waste bag of the Puregraft device, the sample was then weighed and diluted with RL 1:1 (*v*/*v*) at 37 °C (A class). The diluted AT was transiently incubated in a 37 °C incubator (Thermo electron corporation, Waltham, MA, USA) for 15 min before the addition of collagenase enzyme (NB6; Nordmark Biochemicals, Uetersen, Germany) directly into the Puregraft device (injection performed under laminar airflow), at concentrations of 0.10 or 0.25 U/mL for 15, 30, or 45 min at 37 °C under constant agitation using an orbital shaker. Collagenase was previously sampled and validated for free germs. Then, the cell suspension was transferred aseptically into a new medical device (Easyflex+, Macopharma) to allow for cellular concentration by centrifugation (5 min at 400 *g*) (Thermo Fisher Scientific, Waltham, MA, USA) and filtration using a cell strainer with porosity of 200 µm (Becton Dickinson, Franklin Lakes, NJ, USA) to avoid any remaining debris. The filtration and washes to remove residual enzymes were performed with 2:1 (*v*/*v*) RL or saline solution (Baxter) supplemented with 5% of human serum albumin (HAS; LFB Biomedicaments, Courtaboeuf, France) (NaCl 5% HAS). All steps after digestion were aseptically performed in a closed circuit established with subsequent transfers of the cell suspension between transfer bags through sterile connections created using a sterile tubing welder (TSCD; Terumo BCT, Lakewood, CO, USA). Finally, the active substance was concentrated and resuspended with 10 to 15 mL of RL or NaCl 5% HSA. Figure S1 provides images of the process and GMP-compliant criteria.

### 2.4. Inter-Donor Comparative Analysis between Manual and Reference Protocol

Once the LG protocol was developed and validated, six GMP preparations of SVF were performed and retrospectively compared to batches of SVF (n = 33) previously manufactured using the Celution device (Cytori Therapeutics, San Diego, CA, USA); the Celution protocol, used here as the reference protocol, is described in several investigational medical product dossiers for clinical trial authorizations (NCT01813279; NCT02558543; NCT02520843; and NCT02622464). Various biological characteristics of both SVF final products were comparatively assessed and classified as SVF specifications defined for batch release (viability), critical process parameters (recovery yield), and critical quality attributes (cell subset distributions and clonogenic potential) according to the International Conference for Harmonization (ICH; ICH Q5E Harmonized Tripartite Guideline, Comparability of Biotechnological/Biological Products Subject to Changes in their Manufacturing Process; ICH Topic Q 6 B Specifications: Test Procedures and Acceptance Criteria for Biotechnological/Biological Products; and ICH Q8 Harmonized Tripartite Guideline, Pharmaceutical Development). The extraction method for the Celution protocol is provided below.

### 2.5. Celution Protocol for SVF Isolation

SVF was obtained using the Celution 800/CRS automated processing system (Cytori Therapeutics) according to the manufacturer’s instructions. The collected lipoaspirate was washed with RL (Baxter) and enzymatically digested with Celase, a GMP cocktail of enzymes provided with consumables (Worthington Biochemical Corporation, Lakewood, NJ, USA). The cells were concentrated, washed, aseptically recovered, and resuspended in RL.

### 2.6. Intra-Donor Comparative Analysis between the LG and Reference Protocols

**Figure 1:**
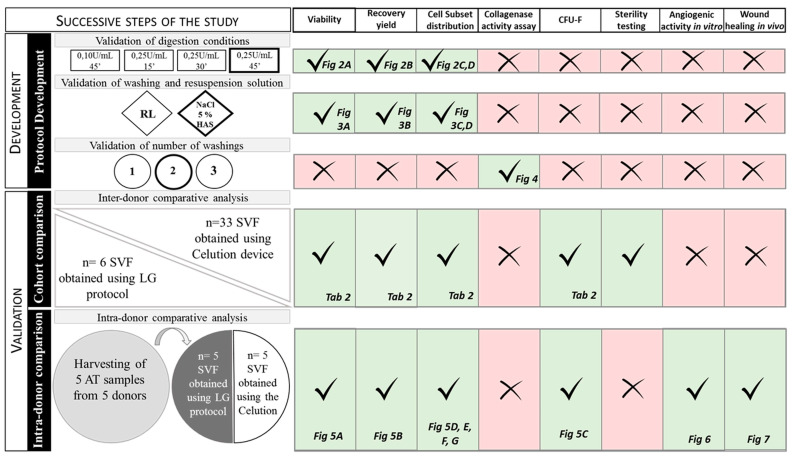
Development steps of the study. The different steps are presented from the top down according to the development chronology. Parameters evaluated at each step are listed, and the resulting figures are indicated. RL: Ringer’s solution. HAS: human serum albumin. SVF: stromal vascular fraction. AT: adipose tissue. Fig: figure; Tab: table. Cross by kareemov1000 from the Noun Project. Check by kareemovic3000 from the Noun Project.2.7. Biological Characterization of SVF.

A comparative analysis between SVF obtained from the LG protocol and the Celution device was also performed according to an intra-donor protocol. AT from the same donor was equally divided in two harvests, and SVF extraction was conducted simultaneously with both protocols before the analysis of SVF characteristics. Figure 1 summarizes the different development steps in this study.

#### 2.7.1. Concentration of Viable Nucleated Cells (VNCs) and Cell Viability

The VNCs concentration and cell viability percentage were determined using a NucleoCounter NC-100 (ChemoMetec, Allerød, Denmark) in duplicate. Recovery yield was calculated as the number of total VNCs obtained divided by the initial volume of AT, which was measured after the removal of infiltration liquid.

#### 2.7.2. Colony-Forming Unit Fibroblast (CFU-F) Assay 

The clonogenic potential of MSCs present in the SVF was assessed by CFU-F assay as follows. First, 500 VNCs were seeded into six-well (Thermo Fisher Scientific) plates in triplicate in proliferation medium [45% Dulbecco modified Eagle’s minimal essential medium, 45% Ham’s-F12, 10% fetal bovine serum (Gibco, Thermo Fisher Scientific), GlutaMAX (100×, Gibco,Thermo Fisher Scientific) gentamicin (Panpharma, Luitré, France), penicillin G (Panpharma), and fungizone (Bristol-Meyers Squibb, New York, NY, USA)]. Then, the colonies were grown for 12 to 14 days, depending on the growth rate of the cells, with medium changes 4 days after seeding, and then every 2 days. At the end of the assay, the culture dishes were rinsed twice with phosphate-buffered saline (PBS; Gibco, Life Technologies, Carlsbad, CA, USA), fixed with abolute ethanol (Cooper, Melun, France), and stained with Giemsa reagent (Sigma-Aldrich, Saint-Quentin-Fallavier, France) for 20 min before two washes with distilled water. Cell colonies were counted under phase-contrast microscopy (DM IL LED; Leica Microsystems, Wetzlar, Germany). All three culture dishes were counted, and the average and standard deviation were calculated to generate the final frequency percentage value, which was expressed as the colony-forming efficiency (total number of colonies) divided by the number of cells seeded × 100.

#### 2.7.3. Flow Cytometry Analysis of SVF Cell Subsets 

Characterization of the SVF cell subpopulations was performed by flow cytometry. Aliquots of 5 × 10^5^ VNCs per tube were suspended in 100 μL of PBS and stained for 20 min at room temperature in the dark with the DRAQ5 nuclear marker and pre-prepared antibody mixes or corresponding isotype controls in matched concentrations. The monoclonal antibody mix included antibodies against CD90, CD146, CD34, and CD45, respectively, which were conjugated with the following fluorochromes: fluorescein isothiocyanate (FITC), phycoerythrin (PE), phycoerythrin-Texas Red-X (ECD), and phycoerythrin-Cyanin 5.1 (PC5) (references listed in Table S1). Red blood cells were lysed in NH_4_Cl for 10 min before the cells were centrifuged and resuspended in PBS Ca^++^/Mg^++^-free medium (Life Technologies). Then, NucBlue (Thermo Fisher Scientific), which allows the discrimination of viable and dead cells, was added for 5 min prior to flow cytometry analysis on a NAVIOS instrument (Beckman Coulter, Brea, CA, USA). Data files were analyzed using Kaluza software (Beckman Coulter) with a multiparameter gating strategy. Briefly, CD45^−^ regenerative cells were discriminated as CD34^−^CD146^+^ pericytes and transitional cells, CD34^+^CD146^+^ endothelial cells (ECs), and CD34^+^CD146^−^CD90^+^ MSCs. The CD45^+^ population includes all hematopoietic cells (macrophages, monocytes, lymphocytes, and neutrophils).

#### 2.7.4. Collagenase Quantification

Washing solutions obtained after all SVF washing steps were collected and sampled to measure the enzyme residual quantity using a collagenase activity assay colorimetric kit (Abcam, Cambridge, UK) according to the manufacturer’s instructions and analyzed on an optical density reader (Tecan, Zürich, Switzerland).

Sterility testing was conducted according to the European pharmacopoeia §2.6.27: microbiological examination of cell-based preparations.

The sterility testing method applied to SVF was previously validated according the ATMP-GMP guidelines §10.4: validation of test methods, by inoculating calibrated commensal bacterial strains of the subcutaneous flora into isolated SVF. Tests were compliant with a detection threshold of 10 colony-forming units.

SVF (150 µL, corresponding to one percent of final volume) was sampled in Bact/alert culture bottles (aerobic and anaerobic culture vials, each containing 40 mL of medium and absorbent resins). The Bact/alert method (Becton Dickinson) uses a computer-controlled incubation/detection system. The media used contained proprietary factors designed to inactivate a wide variety of antibacterial and antifungal agents. Bact/alert culture bottles were incubated at 37 °C and 5% CO_2_ for a total of 10 days, and automated readings were taken every 10 min (according to the European Pharmacopoeia—§2.6.27 Microbiological Examination of Cell-Based Preparations). The detection of organisms resulted in an audible alarm and automatic recording of time of detection.

#### 2.7.5. Tube Formation Assay

SVF samples produced for the intra-donor comparative analysis (n = 2) were loaded at a density of 20,000 cells per well in a μ-slide angiogenesis system (Ibidi, Gräfelfing, Germany) coated with 10 μL of growth factor-reduced Matrigel (6 mg/mL; Corning, Corning, NY, USA) previously polymerized for 30 min; then, they were maintained in endothelial cell growth medium (EGM2-MV; Lonza, Walkersville, MD, USA) at 37 °C and 5% CO_2_. Capillary-like structures were recorded during 96 h using a Leica DMI8 video-imaging inverted microscope equipped with an Incubator I8 (Leica Microsystems) at 5× magnification; images were captured using the Leica Application Suite X software (Las X 3.0.2.16120; Leica Microsystems). The network, length of tubes, and nodes were quantified using ImageJ software (Wayne Rasband, National Institutes of Health, Bethesda, MD, USA) with the Angiogenesis Analyzer plug-in http://image.bio.methods.free.fr/ImageJ/?Angiogenesis-Analyzer-for-ImageJ&lang=en). Each experiment was performed in triplicate.

#### 2.7.6. Spheroid-Based Sprouting Assay

SVF samples produced for the intra-donor comparative analysis (n = 2) were suspended in culture medium containing 0.2% (wt/vol) carboxymethylcellulose (Sigma, Munich, Germany) and seeded in non-adherent round-bottom 96-well plates (Sarstedt, Nümbrecht, Germany), leading to the formation of spheroids with a defined cell number. After 72 h, spheroids were collected and embedded in collagen gel (Collagen I, Rat Tail; Corning). The spheroid-containing gel was rapidly transferred into pre-warmed Lab-Tek II slides (Thermo Fisher Scientific) and allowed to polymerize (30 min); then, 100 μL of EGM2-MV medium was added on the top of the gel. Following 24 h of culture in EGM2-MV medium, spheroids were fixed for 30 min in 4% paraformaldehyde at room temperature. After washing and permeabilization with PBS containing 0.1% Triton X-100 and 1% bovine serum albumin (BSA), for 2 h at 4 °C, spheroids were immunolabeled overnight at 4 °C with phalloidin coupled with Alexa-647 (Thermo Fisher Scientific) (1/100), and nuclei were stained with 6-diamidino-2-phenylindole (DAPI) (1/5000) diluted in PBS containing 1% BSA. After washing, we then captured fluorescent optical image stacks along the *z*-axis at 20× magnification using two lasers in sequential mode under a Leica DMI8 microscope (Leica Microsystems). The Las X software (Leica Microsystems) was used during all image acquisition procedures. Image processing prior to image measurements was performed using Huygens Essential Deconvolution software (Scientific Volume Imaging, Hilversum, The Netherlands) using up to 40 iterations of the classical maximum likelihood estimation algorithm, with a theoretical point spread function and automatic background correction. Then, the images were analyzed using the Sprout Analysis plug-in developed by Eglinger et al. [26] in the Fiji distribution of ImageJ 1.52p (Wayne Rasband) to evaluate the different vascular parameters, such as sprout length and branch points.

#### 2.7.7. In Vivo Experiments 

All experimental procedures were approved by the Region #14 Animal Ethics Committee (reference no. 2015102110259745). A mouse model of cutaneous ischemia was created using 8-week-old female nude Foxn1^nu^ mice. Under general and local anesthesia, the skin and panniculus carnosus muscle of 20 mice were incised in the shape of a square with 3.5-cm sides centered on the mouse back. Vascular subdermal pedicles were dissected, coagulated, and sectioned. The wound edges were fixed with 4-0 polyamide interrupted sutures from either side of a sterile silicone sheet that were 0.5-mm thick and 4 cm per side (Folioxane, Novatech, La Ciotat, France) with a square hole at each center (3 cm per side). This implant was folded and inserted into the incision surrounding the flap to prevent rapid cutaneous revascularization and healing of the edges of the flap. To improve animal welfare conditions, general analgesia by buprenorphine was administered daily for 1 week postoperatively. Four 6-mm punch excisional wounds were made in the dorsal skin of each mouse, according to Sullivan. Silicone donut-shaped splints fashioned from a 0.5-mm thick sterile silicone sheet (Folioxane, Novatech) were fixed to the skin surrounding the wounds with immediate-bonding adhesive and 5-0 nylon sutures (Ethicon, Somerville, NJ, USA), according to Galiano [27]. This cutaneous hypovascularization model was previously validated using three-dimensional computed tomography angiography and laser Doppler flowmetry analyses (data not shown).

Mice were randomly separated into three groups: control group, LG group, and Celution group. Each group received subcutaneous injections of SVF obtained from the same donor with the LG or Celution protocol or a control RL injection. Two million VNCs/cm^2^ were injected at the wound edges of the four punch excisional wounds (0.4 mL of SVF for each wound). Wound closure was measured weekly for 28 days using standardized digital photographs (Canon PowerShot G16, Canon, Paris, France) and analyzed with ImageJ 1.52p. 

#### 2.7.8. Statistical Analyses 

Statistical analyses were performed using Graph Pad Prism 5 (GraphPad Software, La Jolla, CA, USA). Quantitative variables are reported as the mean ± standard deviation (SD) or the median 25–75 th percentile according to the normality of their distributions; minima and maxima are also provided. Continuous variables were compared using Student’s t-test or the nonparametric Mann–Whitney *t*-test. One-way analysis of variance (ANOVA) was employed to compare more than two groups. For assessment of wound closure in the mouse model, data are reported for each group as the average percentage of wound healing ± SD from the four wounds in each mouse. Two-way ANOVA was used for the in vivo wound healing follow-up. A *p*-value < 0.05 was considered to indicate a statistically significant difference.

## 3. Results

### 3.1. Development of the LG Protocol 

#### 3.1.1. Collagenase Digestion Conditions (Time, Concentration)

**Figure 2:**
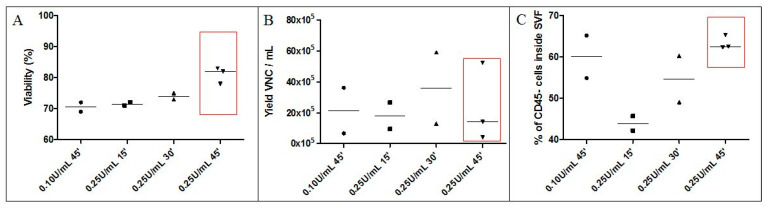
Assessment of several enzyme concentrations and digestion times for enzymatic digestion of AT. Final SVF products obtained with different conditions (0.10 U/mL and 45 min; 0.25 mL and 15 min; 0.25 U/mL and 30 min; and 0.25 U/mL and 45 min) were assessed in terms of (**A**) viability, (**B**) yield of VNCs per mL AT, and (**C**) cell subset distribution including the percentage of CD45^−^ regenerative cells. The red frame shows the chosen condition. Experiments were reproduced one to three times according to the available AT volume. SVF: stromal vascular fraction; VNC: viable nucleated cell; ’: minutes.

As shown in Figure 2A, the use of collagenase at concentrations of 0.10 U/mL AT for 45 min or 0.25 U/mL AT for 15 or 30 min at 37 °C yielded SVF with median cell viabilities of 70.50%, 71.50%, and 74.00%, respectively, which are below the 80% threshold, which is a value approved in several investigational medicinal product dossiers by the French national medecines safety agency (cf. Methods for NCT numbers). The median cell viability of SVF obtained from collagenase digestion at 0.25 U/mL AT for 45 min reached 81%.

Under these conditions, the yield of VNCs/mL AT ranged from 4.00 × 10^5^ to 59.2 × 10^5^ with a median value of 11.2 × 10^5^ VNCs/mL AT (Figure 2B). The median VNCs recovery values were 21.52 × 10^5^; 18.07 × 10^5^; 36.10 × 10^5^; and 14.10 × 10^5^ per mL AT when collagenase digestion was performed under conditions of 0.10 U/mL and 45 min; 0.25 U/mL and 15 min; 0.25 U/mL and 30 min; and 0.25 U/mL and 45 min, respectively.

Flow cytometry analyses of the percentage of CD45^−^ regenerative cells within SVF are shown in Figure 2C. The median proportion of CD45^−^ regenerative cells was the lowest at 43.94% for the 0.25 U/mL and 15 min condition. The median percentage of CD45^−^ regenerative cells for conditions of 0.10 U/mL and 45 min, and 0.25 U/mL and 30 min were 60.10% and 54.71%, respectively. Finally, the highest percentage (62.55%) was obtained under conditions of 0.25 U/mL and 45 min. 

Altogether, the 0.25 U/mL and 45 min condition was adopted, as it allows SVF to comply with the usual viability specification with acceptable yields for VNCs and the proportion of regenerative cells.

#### 3.1.2. Washing Solution and Final Excipient for SVF Cells

**Figure 3:**
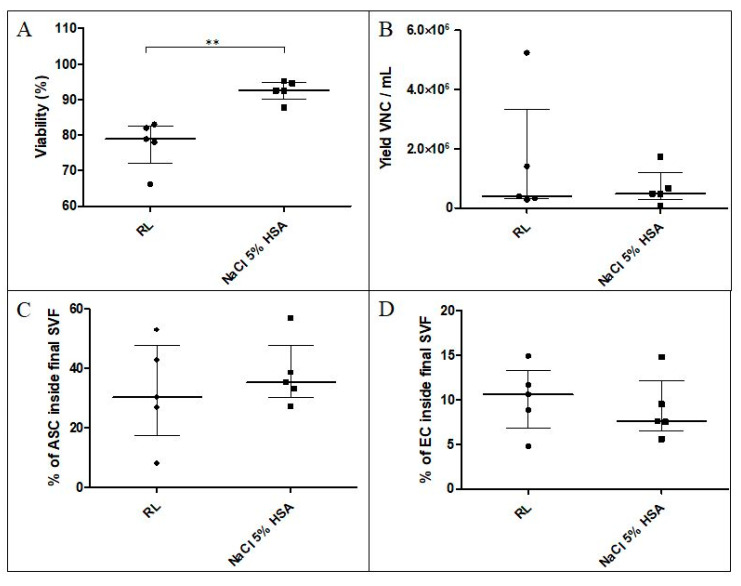
A ssessment of two different solutions for filtration, washing, and final resuspension of SVF. The solutions tested were RL and NaCl 5% HSA. (**A**) Viability was statistically higher for the NaCl 5% HSA group (*p* = 0.01). (**B**) Yield of VNCs per mL AT, (**C**) percentage of MSCs, and (**D**) ECs were not statistically different. RL: Ringer’s lactate; NaCl 5% HSA: saline solution enriched with HAS; VNCs: viable nucleated cells; MSCs: mesenchymal stem cells; ECs: endothelial cells.**: *p* = 0.01.

The obtained SVF cell suspension was filtered, washed three times, and resuspended in RL (n = 5) or NaCl 5% HSA (n = 5). The measured cell viability of the finished product was statistically increased in the NaCl 5% HSA group compared to the RL group (median 92.50% (90.10–94.90%) vs. 78.90% (72.10–82.50%), *p* = 0.01) (Figure 3A). The amount of VNCs recovered per mL AT did not statistically differ between the two groups (Figure 3B). However, recovery was highly variable in the RL group, ranging from 2.80 × 10^5^ to 52.4 × 10^5^ VNCs/mL AT with a median value of 4.00 × 10^5^ VNCs/mL AT (2.98–33.20 × 10^5^ VNCs/mL AT) and a variation coefficient of 139.27%. Conversely, the yield was more repeatable with the NaCl 5% HSA washing solution, with a median value of 4.84 × 10^5^ VNCs/mL AT (2.79–11.93 × 10^5^ VNCs/mL AT) and a variation coefficient of 89.9%. Proportions of MSCs (Figure 3C) and ECs (Figure 3D) identified by flow cytometry within the final active substance did not statistically differ between the two groups.

Thus, NaCl 5% HSA was chosen as the main excipient for filtering and washing of the isolated SVF due to the high viability it confers to the cell product and its ability to maintain its viability (data not shown).

#### 3.1.3. Validation of the Necessary Number of Washings 

**Figure 4:**
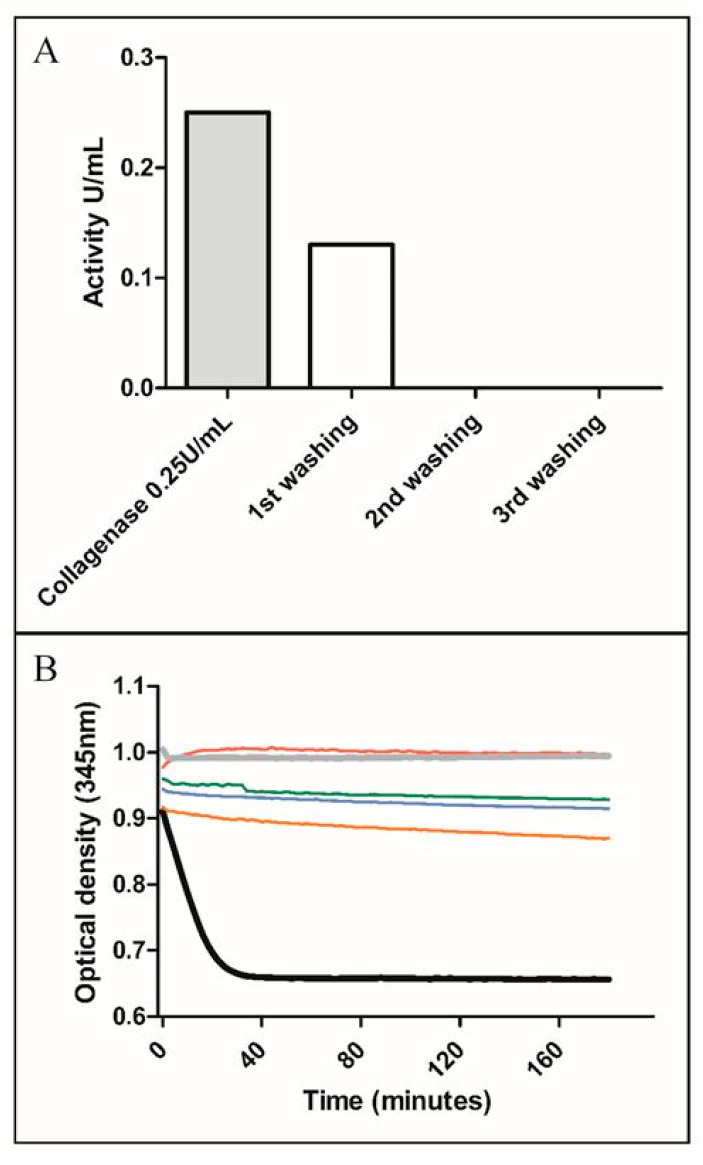
Kinetics of substrate degradation by collagenase. (**A**) A representative experiment of four enzyme activity assay replications: collagenase was undetectable from the second washing. (**B**) The optical density reflects degradation of the substrate by collagenase over time. The slope of the regression line was calculated for the second washings of four SVF preparations: SVF-A, SVF-B, SVF-C, and SVF-D, respectively: −0.00003, −0.0001, −0.0001, and −0.0002; slope of the positive control: −0.0113; slope of the negative control: −0.00002.

Four independent SVF batches (A–D) were produced using the parameters validated above. After each centrifugation/washing step, the washing supernatants were retained to quantify collagenase concentrations. Analysis of the kinetics of substrate degradation by collagenase over 180 min indicated that collagenase was undetectable when at least two washing steps were applied (Figure 4A). Linear regression based on optical density evolution over time demonstrated that each of the second washing assays provided a slope of the regression line inferior to 0.0005, which reflected that collagenase was non-measurable from this point. Details of each experiment are provided in Figure 4B. The slope of the regression line of substrate degradation by the positive control, corresponding to collagenase at 0.25 U/mL, was equal to −0.0113, whereas the slope of the negative control was −0.00002. The slope of the second washing solutions of SVF-A, SVF-B, SVF-C, and SVF-D were −0.00003, −0.0001, −0.0001, and −0.0002, respectively. This indicates that after digestion, SVF can be washed only twice to permit clinical use with an undetectable residual quantity of collagenase.

Collectively, these results identify production parameters that allowed us to optimally design the LG protocol and comparatively evaluate it with reference to the Celution-based method. Table 1 summarizes the differences in the SVF manufacturing process using the LG and Celution protocols.

### 3.2. Comparability of LG and Celution-Based Protocols for SVF Manufacturing 

#### 3.2.1. Inter-Donor Comparative Analysis of SVF Produced using the Celution Device or LG Standardized Protocol

The viability of nucleated cells manufactured with the LG protocol (94.15% (89.83–93.03%)) was significantly higher compared to that acquired from the Celution device (87.30% (84.60–90%), *p* < 0.01). Similarly, the LG protocol generated a greater number of VNCs/mL AT, with a median value of 4.20 × 10^5^ (3.35–10.58 × 10^5^ VNCs/mL AT) *vs.* 2.52 × 10^5^ (1.50–3.18 × 10^5^ VNCs/mL AT) in the Celution group (*p* < 0.01). The clonogenic potential of MSCs, assessed via CFU-F assay, did not statistically differ between the two processes (*p* = 0.43). The distribution of various cell subpopulations of SVF from the LG protocol was similar to that of SVF from the reference protocol, as the respective proportions of MSCs, ECs, pericytes, and leukocytes did not statistically differ between the two protocols. The results are summarized in Table 2.

#### 3.2.2. Intra-Donor Comparative Analysis of SVF Produced Using the Celution Device or Final LG Protocol

**Figure 5:**
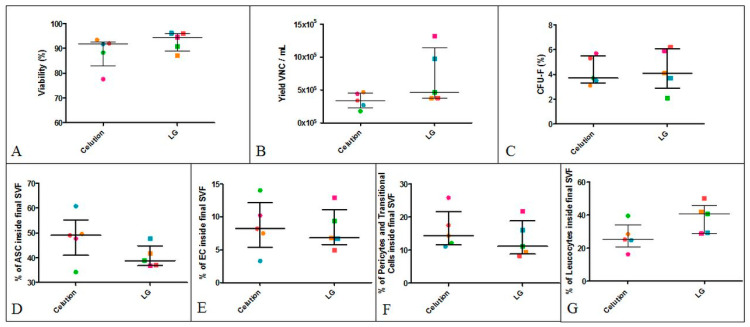
Intra-donor comparative analysis of SVF produced using the Celution device or final LG protocol. Five SVF preparations were produced from five donors with the Celution device or LG protocol. Each color represents a donor. (**A**) Viability (*p*-value = 0.3090), (**B**) yield of VNCs per mL AT (*p*-value = 0.1256), (**C**) CFU-F (*p*-value = 0.7790), (**D**) % of ASCs (*p*-value = 0.0722), (**E**) % of ECs (*p*-value = 0.7610), (**F**) % of pericytes and transitional cells (*p*-value = 0.2874), and (**G**) % of leukocytes (*p*-value = 0.0517) were evaluated.

No significant difference was observed in the specifications of the comparatively analyzed SVF: the median percentage of viability for the LG protocol was 94.50% (88.95–96.05%) versus 91.70% (82.95–92.70%) for the reference protocol (Figure 5A). The LG protocol generated a median recovery value of 4.64 × 10^5^ VNCs/mL AT (3.74–11.45 × 10^5^ VNCs/mL AT) vs. 3.39 × 10^5^ VNCs/mL AT (2.25–4.54 × 10^5^ VNCs/mL AT) with the Celution device (Figure 5B). The clonogenic potential of MSCs was 4.10% (2.90–6.05%) for the LG protocol vs. 3.70% (3.30–5.50%) for the Celution device (Figure 5C). The cell subset distributions of SVF produced with the LG and Celution protocols were similar: ASCs were the major population with 38.80% (36.85–44.75%) vs. 49.00% (40.95–55.15%), whereas ECs represented only 6.80% (5.80–11.10%) vs. 8.20% (5.40–12.10%). Pericytes and transitional cells accounted for 11.20% (8.87–18.90%) vs. 14.41% (11.65–21.70%). A non-significant difference was observed for leukocytes, with 40.60% (29.00–45.90%) vs. 25.20% (20.50–34.00%) for the LG and Celution protocols, respectively. These results showed that SVF manufactured according to the LG process contains similar proportions of regenerative cells compared to the reference. The only slight difference observed concerned leukocytes, which tended to be higher in SVF obtained with the LG protocol.

#### 3.2.3. Sterility Testing

Samples of SVF (n = 3) produced using the LG protocol were submitted to sterility testing; the results established that no microorganisms were present.

#### 3.2.4. Analysis of the SVF Angiogenic Capacity In Vitro 

**Figure 6:**
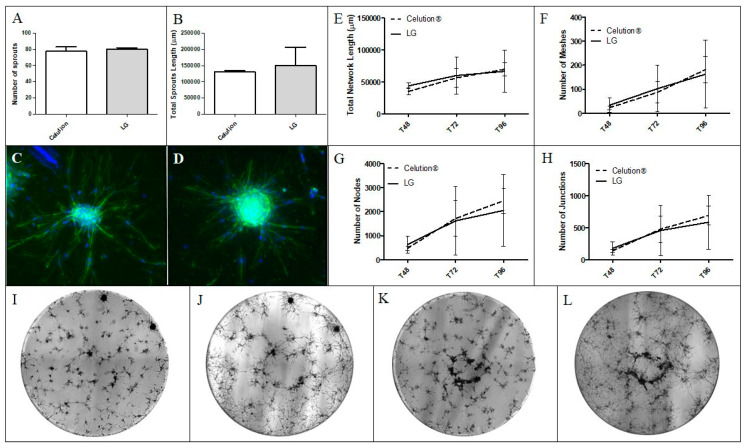
Representative experiment of the spheroid-based assay and pseudotube formation in Matrigel of SVF produced with the LG or reference protocols. SVF was produced from the same AT sample with both processes (n = 2). After 24 h of culture, spheroids were labeled with 6-diamidino-2-phenylindole (DAPI) (nucleus) and phalloidin (actin cytoskeleton), which allowed measurement of (**A**) the number of sprouts and (**B**) total sprout length in µm. Representative pictures of one spheroid of the (**C**) Celution group and (**D**) the LG group were taken at 20× magnification. Evolution of the network was followed after seeding 20,000 cells per well coated with Matrigel. Several parameters were evaluated: (**E**) total network length, (**F**) number of meshes, (**G**) number of nodes, and (**H**) number of junctions. Representative images of pseudotube formation by (**I**) Celution SVF at 72 h and (**J**) 96 h post seeding, and by (**K**) LG SVF at 72 h and (**L**) 96 h post seeding were taken at 4× magnification.

The angiogenic capacity of SVF manufactured using both protocols was compared. Using the spheroid-based assay, the number of sprouts and total sprout length were equivalent between the LG and Celution-based protocols (80.00 (78.00–82.00) and 1.50 × 10^5^ µm (0.94–2.06 × 10^5^ µm) vs. 77.50 (72.00–83.00) and 1.31 × 10^5^ µm (1.27–1.34 × 10^5^ µm), respectively) (Figure 6A,B). The kinetics of pseudotube formation in Matrigel matrix were also similar between the two groups; network formation started at ≈48 h post seeding and reached peak values at ≈96 h (t96). The median total network length at t96 was 6.64 × 10^4^ µm for the LG group and 6.96 × 10^4^ µm for Celution group (Figure 6E). The number of meshes at t96 was 162.50 for cells obtained with the LG protocol and 181.50 for Celution (Figure 6F). The numbers of nodes were 2051.50 and 2453.00 for SVF produced by the LG and Celution processes, respectively (Figure 6G). Finally, the number of junctions in the LG group was 585.50 vs. 688.00 in the Celution group (Figure 6H). These results indicate that SVF products from both manufacturing protocols are comparable in terms of angiogenic potential in vitro.

#### 3.2.5. Analysis of the Angiogenic Capacity In Vivo

**Figure 7:**
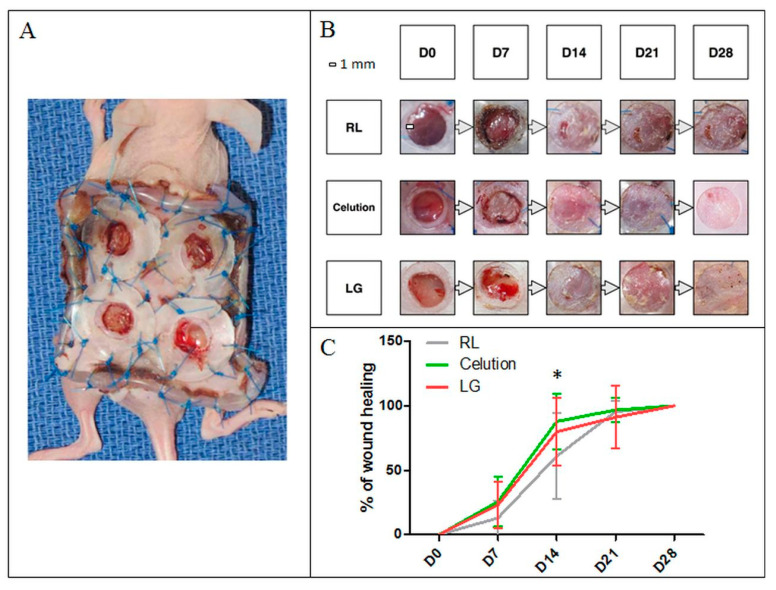
Testing of the angiogenic ability of SVF produced with both protocols in a mouse model of subcutaneous ischemia versus placebo. SVF was produced from the same AT sample (n = 1) with both processes and injected into the wound edges of six mice per group. (**A**) Representative image of the mouse model of subcutaneous ischemia on day 0 (D0). The control consisted of RL in injections in four mice. Wound healing was quantified at D7, D14, D21, and D28 post-injection by analysis of standardized images. (**B**) Representative images of wound closure for each group and for each time. (**C**) The kinetics of wound healing did not differ between the LG and Celution groups. On D14, wound healing was significantly greater in the LG and Celution groups compared to placebo, with respective *p*-values < 0.05 and <0.001. No difference was observed between the three groups at D7, D21, and D28.*: *p* < 0.05.

SVF produced for the intra-donor comparative analysis was tested for its wound-healing ability (LG: n = 1; Celution: n = 1) in a mouse model of subcutaneous ischemia inspired by the Galiano model [27]. Three groups of mice received injections of SVF produced by the LG protocol or Celution device. RL injections were used as a control (Figure 7). Mice that received an injection of SVF healed faster compared to the control group, with an average wound healing at day 7 (D7) of 23.17% (±17.96%) for the LG group (*p* > 0.05) and 25.70% (± 19.35%) for the Celution group (*p* > 0.05) vs. 12.99% (±13.39%) for the placebo group. The same conclusion was observed at D14, with an average wound healing of 79.98% (± 26.50%) and 87.96% (±21.71%) for mice who received SVF from the LG (*p* < 0.05) or Celution protocol (*p* < 0.001) vs. 61.08% (± 33.67%) for placebo. At D21, the percentages of wound healing were 95.56% (± 8.20%), 96.80% (± 9.41%), and 91.16% (± 24.49%) for the control, Celution, and LG groups, respectively; no statistically significant difference was observed. At D28, all wounds were healed.

## 4. Discussion

In this study, we developed and validated a protocol, based on a classical enzymatic digestion using collagenase [25], that was optimized to allow the manufacturing of clinical grade SVF, and it can be proposed as an attractive alternative to the use of automated medical device-based methods. The risk analysis performed between LG and Celution protocols was favorable to LG protocol for two reasons: (i) a perfect control over the quality of raw materials and (ii) the compliance to European regulation (Table S2). Indeed, this protocol fully complies with GMP requirements, as manufacturing under grade A in B cleanroom conditions as soon as open steps are required, and it uses only media and materials suitable for therapeutic use. Futhermore, LG protocol presents the avantage of being less invasive as a lower quantity of AT harvesting is needed compared to Celution protocol (at least 50 mL versus 100 mL). Thus, this procedure could be addressed to lower body-mass index patients.

We defined each step of the process by evaluating changes in manufacturing parameters that are critical both for regulatory requirements and potential effectiveness of the final medicinal product. Viability of the nucleated cells recovered after AT enzymatic digestion was chosen to be the only product specification, as that should confirm the quality and ensure the potential efficacy of the final product according to ICH guidelines. The yield of VNCs per mL AT was defined as a critical process parameter as it is the most representative of isolation protocol efficiency. In addition, it may have an impact on the product quality and should be controlled according to ICH guidelines. The applicability of SVF isolation methods is sometimes hampered by the poor availability of large volumes of AT as the starting biological material in patients with a low body mass index such as those presenting with vascular and/or inflammatory chronic disease. The use of a recommended AT volume of ≈100 mL in the Celution procedure can be viewed as a potential limitation. Therefore, optimization of the SVF yield is critical, making monitoring of the digestion temperature one of the most crucial parameters to obtain an effective digestion. Manual methods for SVF isolation allow digestion to be directly run in a 37 °C thermostatic chamber. Finally, the cell subset distribution and clonogenic potential of MSCs are considered critical quality attributes according to ICH guidelines. Thus, although the mechanism of action of the SVF is not fully established, and no study to date has reported a link between the cell subset distribution of SVF and its clinical benefit in patients, it has been established that the MSC counterpart plays a prominent role through its paracrine activities and/or multipotent differentiation ability [28]. MSCs from AT have been shown to exert immunomodulatory properties through the secretion of various growth factors and cytokines [29,30] and have also been investigated for their anti-fibrosis effects [31]. Furthermore, the synergistic actions involving different CD45^−^ SVF cells are also crucial for pro-healing and angiogenesis, as ECs and pericytes make direct contributions to vessel formation and maturation [32,33] and paracrine support of MSC function [10,34,35]. For these non-exhaustive reasons, it appears essential to guarantee that our final SVF product intended for use in various clinical contexts is composed of a sufficient proportion of regenerative cells and retains significant MSC clonogenic potential to ensure the desired product quality.

Based on these parameters, the selected protocol consists of an AT digestion step with collagenase at 0.25 U/mL AT for 45 min. The best cell viability was achieved by using the highest concentration and the longest incubation time for collagenase. Consistently, as reported by Lee and colleagues, SVF viability was shown to decrease with increasing treatment duration, but this effect is significant only for a duration equal to or greater than 60 min [36]. The following washing steps were defined using NaCl 5% HSA, allowing for higher SVF viability compared to RL, which was used in the Celution protocol, and ensuring an undetectable residual quantity of collagenase. The approximate duration for SVF production using the LG protocol is three and a half hours.

In the second part of our work, we first retrospectively compared the final product manufactured with the LG protocol to a reference process based on use of the Celution automated device, which was the first commercially available device for SVF production following the discovery of multipotent stem cells in AT by Zuk and her team [3]. Interestingly, the viability and recovery yield were significantly higher in the LG protocol compared to biological data from SVF obtained with the Celution device as well as data provided in the different investigational medicinal product dossiers established for the regulatory approval of clinical trials. One hypothesis is that composition differs between collagenase (enzyme used in the LG protocol) and Celase (enzyme used in the Celution) and could have an impact on the recovery yield and the viability of SVF. Furthermore, the quality control methods remained unchanged compared to the reference, and sterility testing showed that the LG protocol produced a sterile end product for all batches manufactured.

More importantly, the robust intra-donor comparison performed on five batches from the same AT harvest and discriminated according to the LG or Celution-based process allowed us to attest the comparability of the two methods. The viability, yield of VNCs per mL AT, CFU-F assay, and cell subset distribution were statistically similar between both cell suspensions. One trend was a higher percentage of leukocytes within SVF obtained with the LG protocol. This could be explained by the automated initial washing step of the AT provided by the Celution device. This automated washing takes into account the level of blood contamination of the initial AT and is supposed to be more effective, as rinsing and washing steps are repeated until obtaining non-bloody AT. In the LG protocol, the initial washing step is defined by three rounds of washing with RL in 1:1 proportion with AT, leading to an acceptable visual aspect of the AT before digestion. No further development was performed on this initial step, as collagenase activity is driven by required electrolytes present in RL.

To our knowledge, our study is the first to implement in vitro angiogenesis testing to analyze the comparability of SVF manufacturing methods. The expected added value is to provide functional testing that can be evaluated in the future as a potency assay. Such testing is still not defined and remains challenging in the context of the use of SVF as an experimental ATMP product. Our results showed that the sprouting ability as well as the capacity to self-organize into capillary-like structures in a Matrigel matrix in vivo was not different between the two SVF production methods. This observation is in line with the fact that the main cell subsets supporting SVF angiogenic activity were similarly represented in SVF from both processes. This also suggests that the functional paracrine interactions between SVF cells during capillary formation are equivalent regardless of the SVF manufacturing process. Consistently, both SVF products tested in an innovative wound model of cutaneous excision and ischemia in nude mice were able to accelerate wound closure. The pro-healing activity did not significantly differ between SVF obtained from the LG or Celution protocols.

In the few last years, a substantial number of automated and semi-automated devices have been introduced or are currently in development, with a total of 17 devices reported in the review of Oberbauer and colleagues [37]. A large body of articles have reported the biological characteristics of these devices [38,39,40], and in some cases, they use a manual method as reference [41,42]. However, the described manual protocols are not associated with any development data, leading to a high technical heterogeneity in the different steps of the processes and, consequently, in the biological results. In view of this literature, the LG protocol displays interesting characteristics in terms of viability and cell recovery [43]. As illustrated in Table 3, gathering manufacturing data from 12 studies and 20 SVF preparations, only one study showed higher viability than SVF obtained with the LG protocol, and 14 productions presented lower recovery yields than those achieved with the LG protocol. In addition, the estimated costs for one SVF production using the LG protocol under the ATMP-hospital exemption include consumables for harvesting and extraction (around USD 1600); staff costs (around USD 1500); facilities management (around USD 650); and biological quality controls (around USD 1200) for a total of USD 5000. Conversely, Aronowitz and colleagues [44] reported that automated devices cost over USD 50,000, which is associated with a significant burden due to expensive single-use kits that can cost hundreds or thousands of dollars. Furthermore, some of these devices are not fully closed systems, meaning that one of the steps must be performed in an open environment, which is not compatible with the full application of GMP guidelines. Indeed, these guidelines prescribe that all open steps are only tolerated if performed under grade A laminar airflow, limiting the use of these devices, which are too cumbersome to be placed under a microbiological safety station. However, it should be noted that the Celution is one of the most expensive and can also be the most cumbersome of these devices. It would be interesting to carry out future comparative studies between the LG protocol and other automated devices, after having duly checked their GMP compliance, as it could provide better reproducibility compared to a manual method.

In conclusion, this study reports for the first time the development of a GMP-compliant process for manual SVF isolation. This process showed satisfactory performance criteria and allows the manufacturing of clinical-grade SVF with angiogenic and pro-healing potency in vivo comparable to that obtained with an automated device previously used in clinical trials. Although future studies are required to evaluate the SVF obtained using this validated protocol in clinical settings, our study offers an easy to implement, cost-effective, and standardized technical option that is part of a global strategy aimed at promoting SVF-based cell therapy and favoring patients’ access to medical innovations.

## Figures and Tables

**Table 1:** Differences between LG and Celution protocols. LG: the developed SVF production process, named the person who originally developed it. NA: not applicable.

	Celution Protocol		LG Protocol	
AT harvesting	Using Khouri cannula		Using Khouri cannula	
AT repackaging	Packaging in 60-mL Luer Lock syringes		NA	
Device preparation	Tensioning Celution device, settlement of consumables, and seal check		NA	
AT transfer	AT transfer in Celution device		AT transfer in Puregraft device	
Settling and discarding infiltration liquid	Automated	Visual check of discarding infiltration liquid	Discarding infiltration liquid performed using Puregraft device	
Weighing	Automated	At least 100 mL of AT needed	NA	
AT washing	Automated	From two to five washings with RL and visual check	Three washings using RL	
Weighing	Automated	No particular volume required	AT volume between 50 and 250 mL	
Dilution	NA		Two-fold dilution with RL	
Incubation	NA		Incubation of the diluted AT for 15 min at 37 °C	
Enzyme preparation	Celase reconstitution		Collagenase thawing (previously reconstituted at 20 U/mL)	
Digestion	Automated	Injection of 5 mL of reconstituted Celase into Celution device	Injection of collagenase at 0.25 U/mL into Puregraft device for 45 min at 37 °C under agitation	
Concentration	Automated	Visual check of the concentration	Closed Circuit	Cellular concentration by centrifugation (400 *g*, 5 min)
Filtration	NA		Closed Circuit	Cell strainer 200-µm porosity
Washings	Automated	With RL	Closed Circuit	Two washings with NaCl 5% HSA
Collection of SVF	SVF resuspended in RL		SVF resuspended in NaCl 5% HSA	

**Table 2:** Inter-donor comparative analysis of SVF produced using the Celution device or LG protocol. Detailed data of the 33 SVF products obtained with the Celution reference protocol and the six SVF products obtained with the LG protocol. P-values were obtained after average comparison using a nonparametric test.

	Viability		Yield of VNCs (×10^5^/mL AT)		CFU-F		ASC		EC		Pericytes and Transitional Cells		Leukocytes (Macrophages, Neutrophils, and Lymphocytes)	
	LG Protocol	Celution Device	LG Protocol	Celution Device	LG Protocol	Celution Device	LG Protocol	Celution Device	LG Protocol	Celution Device	LG Protocol	Celution Device	LG Protocol	Celution Device
Average	93.05%	87.00%	6.30	2.50	4.05%	4.80%	39.50%	43.99%	8.40%	7.66%	12.70%	10.29%	39.50%	38.06%
Standard deviation	3.50%	4.00%	4.20	1.23	1.70%	1.70%	4.70%	11.49%	2.80%	6.15%	5.20%	7.95%	8.80%	12.45%
Median	94.15%	87.30%	4.20	2.52	3.92%	5.00%	37.90%	44.00%	8.07%	6.00%	10.64%	8.00%	41.25%	37.00%
25% Percentile	89.83%	84.60%	3.35	1.50	2.24%	3.42%	36.25%	36.50%	6.25%	5.00%	8.67%	3.00%	29.15%	31.50%
75% Percentile	96.03%	90.00%	10.58	3.18	5.97%	5.90%	43.31%	49.50%	10.43%	8.50%	17.45%	14.50%	47.50%	49.00%
Minimum	87.20%	77.60%	3.20	0.77	2.07%	1.30%	34.90%	25.50%	4.90%	2.70%	8.30%	1.00%	28.70%	12.70%
Maximum	96.10%	93.40%	13.20	5.76	6.20%	8.00%	47.70%	81.40%	12.90%	37.10%	21.80%	32.20%	49.90%	63.60%

**Table 3:** Yield recovery and viability from 12 SVF extraction studies. Studies that reported both viability and yield recovery after enzymatic digestion were selected. The reference of the study is provided in the first column. The method of SVF extraction is reported in the second column; if an automated or medical device was used, it is specified. The third column shows the number of batches on which the results were evaluated. The two last columns indicate the percentage of viability and yield recovery per mL of AT. It is important to note that the determination of viability and recovery yield were not conducted by the same method in each study. Comparison with the LG protocol was conducting using the inter-donor comparison value.

Studies	SVF Extraction Method	Number of Batches	Viability (%)	Yield of VNCs (×10^5^/mL AT)
[45]	Non-automated	18	93.90	4.00
[46]	Non-automated	44	>50.00	3.08
[42]	Non-automated	11	>90.00	1.60
	Sepax		>90.00	2.60
[41]	Non-automated	6	82.40	7.01
	Icellator		80.70	7.02
[47]	Celution	5	93.00	2.40
	Lipokit		72.00	0.35
	PNC Multistation		57.00	1.70
	CHA-Station		87.00	0.05
[48]	Celution	31	87.70	3.60
[49]	Non-automated	130	85.05	1.81
[50]	Non-automated	9	80–90	2.30
[51]	GID SVF1	52	83.00	7.19
[52]	Non-automated	11	96.00	2.05
[39]	Non-automated	4	75.80	7.95
	GID SVF1		81.47	4.25
	Non-automated (Puregraft)		77.45	2.50
	Stem.pras with Duografter		69.30	5.35
[53]	Non-automated	19	82.20	1.85

## Supplementary Materials

The following are available online at https://www.mdpi.com/2073-4409/9/10/2158/s1, Figure S1: Illustration of the LG protocol and GMP compliance inputs, Table S1. References for antibodies used in SVF phenotypical characterization. Table S2: Risk analysis between Celution and LG processes.
