# Supplementary material for: Development and Validation of a Fully GMP-Compliant Process for Manufacturing Stromal Vascular Fraction: A Cost-Effective Alternative to Automated Methods
Source: Cells. 2020 Sep 24;9(10):2158. doi: 10.3390/cells9102158 (PMC7598595; doi:10.3390/cells9102158)
Supplement: Supplementary file 1 [file cells-09-02158-s001.zip › cells-935250 supplementary/Supplementary materials .docx]

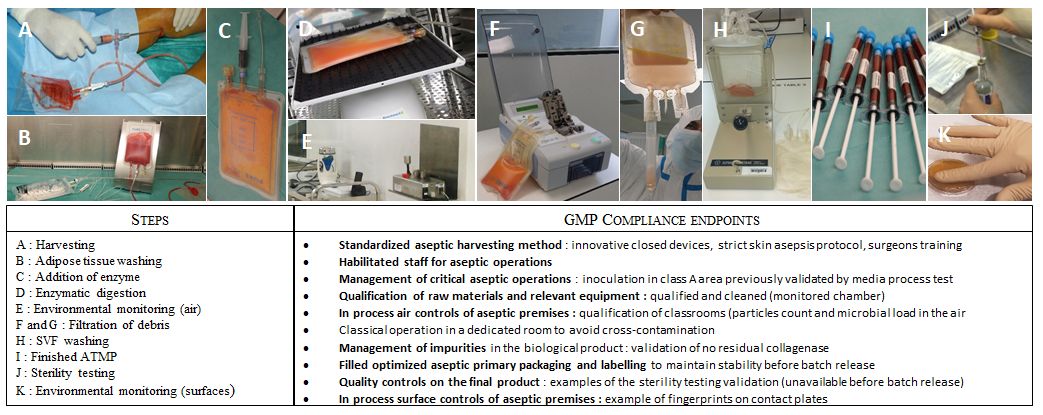
Supplementary Materials:

**B**

**C**

**D**

**F**

**G**

**H**

**I**

**J**

**K**

Figure S1: Illustration of the LG protocol and GMP compliance inputs.

| **Specificity** | **Fluorochrome** | **Supplier** | **Reference** | **Volume** |
| --- | --- | --- | --- | --- |
| CD90 | Fluorescein isothiocyanate (FITC) | Beckman Coulter | IM1839U | 10 μL |
| CD146 | Phycoerythrin (PE) | Beckman Coulter | PN A07483 | 10 μL |
| CD34 | Phycoerythrin-Texas Red-X (ECD) | Beckman Coulter | IM2709U | 10 μL |
| CD45 | Phycoerythrin-Cyanin 5.1 (PC5) | Beckman Coulter | A07785 | 10 μL |
| DRAQ5 | NA | eBioscience | 65-0880-96 | 10 μL (diluted 25-fold) |
| NucBlue | NA | Thermo Fisher Scientific | R37606 | 5 μL |

**Table S1. References for antibodies used in SVF phenotypical characterization.** NA: Not applicable.

| **Risks** | **Risk management actions** | | **Risk level** | | **Conclusion** |
| --- | --- | --- | --- | --- | --- |
|  | Celution | LG | Celution | LG |  |
| Lack of control over the quality of raw materials (consumables) | - Little control on the supplier  - Inspection upon receipt of consumables | - Possibility of auditing supplier  - Inspection upon receipt of consumables  - Quality control on the enzyme | High risk | Low risk | **Lower risk for the LG protocol** |
| Lack of control over the quality of adipose tissue collection | - Enhanced skin asepsis at the sampling site  - Sampling performed by experienced senior surgeons | | Moderate risk | Moderate risk | **Equivalent risk** |
| Lack of quality control of equipment: equipment / device | Annual audit | Annual critical equipment qualification | Moderate risk | Low risk | **Lower risk for the LG protocol** |
| Lack of control of the manufacturing process | Enzymatic digestion efficiency | | Moderate risk | Low risk | **Lower risk for the LG protocol** because use of a monitored thermostatic chamber |
|  | Reproducibility data and validation data | | Low risk | Moderate risk | **Lower risk for the Celution protocol** because he experience gained with the Celution process is older and more important in terms of number of batches produced |
|  | Aseptic validations (media process test) | | Low risk | Low risk | **Equivalent risk** |
| Lack of control over the quality of the active substance and finished product | Comparability data | | Low risk | Low risk | **Equivalent risk** |
| Lack of control of environmental risk | Use of the device in class B (semi-open system) : not compliant | Semi-open phases in class A and closed phases in class B : compliant | High risk | Low risk | **Lower risk for the LG protocol** |

**Table S2: Risk analysis between Celution and LG processes.**  The risk analysis was performed by the person in charge of quality control and validated by the French national medicines safety agency as part of a comparability study.
