# Supplementary figures and images for: Development and Validation of a Fully GMP-Compliant Process for Manufacturing Stromal Vascular Fraction: A Cost-Effective Alternative to Automated Methods
Source: Cells. 2020 Sep 24;9(10):2158. doi: 10.3390/cells9102158 (PMC7598595; doi:10.3390/cells9102158)

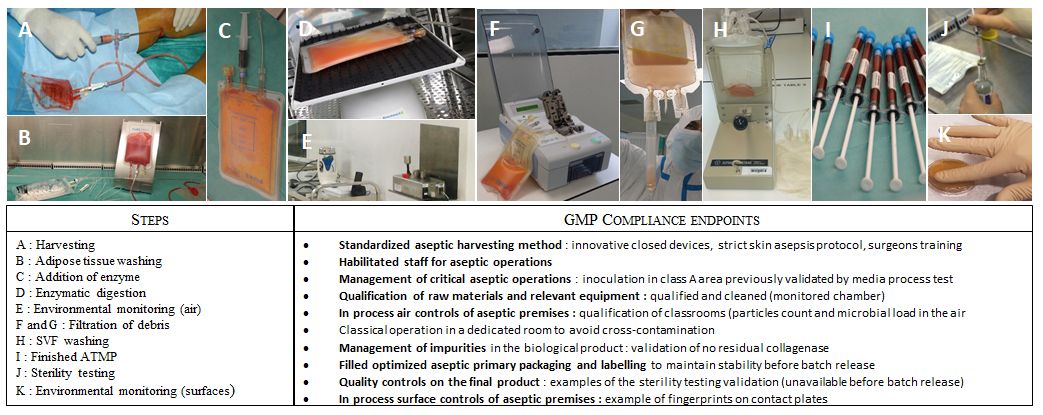

Supplement: Supplementary file 1 [file cells-09-02158-s001.zip › cells-935250 supplementary/figS1.jpg]
